# A natural human monoclonal antibody targeting Staphylococcus Protein A protects against *Staphylococcus aureus* bacteremia

**DOI:** 10.1371/journal.pone.0190537

**Published:** 2018-01-24

**Authors:** Avanish K. Varshney, Galina A. Kuzmicheva, Jian Lin, Kevin M. Sunley, Rodney A. Bowling, Tzu-Yu Kwan, Heather R. Mays, Anu Rambhadran, Yanfeng Zhang, Rebecca L. Martin, Michael C. Cavalier, John Simard, Sushma Shivaswamy

**Affiliations:** XBiotech USA Inc., Austin, Texas, United States of America; Scripps Research Institute, UNITED STATES

## Abstract

*Staphylococcus aureus* can cause devastating and life-threatening infections. With the increase in multidrug resistant strains, novel therapies are needed. Limited success with active and passive immunization strategies have been attributed to *S*. *aureus* immune evasion. Here, we report on a monoclonal antibody, 514G3, that circumvents a key *S*. *aureus* evasion mechanism by targeting the cell wall moiety Protein A (SpA). SpA tightly binds most subclasses of immunoglobulins via their Fc region, neutralizing effector function. The organism can thus shield itself with a protective coat of serum antibodies and render humoral immunity ineffective. The present antibody reactivity was derived from an individual with natural anti-SpA antibody titers. The monoclonal antibody is of an IgG3 subclass, which differs critically from other immunoglobulin subclasses since its Fc is not bound by SpA. Moreover, it targets a unique epitope on SpA that allows it to bind in the presence of serum antibodies. Consequently, the antibody opsonizes *S*. *aureus* and maintains effector function to enable natural immune mediated clearance. The data presented here provide evidence that 514G3 antibody is able to successfully rescue mice from *S*. *aureus* mediated bacteremia.

## Introduction

*Staphylococcus aureus* is found in the nose and on the skin of 25–30% of healthy adults[1]. However, in a person with a weakened immune system, if it manages to breach the periphery, *S*. *aureus* infections can be serious or even fatal[2–4]. Fatal infections are often associated with bacteremia, which, before the widespread use of antibiotics, had a 65–70% mortality rate. Now, even with best practices and latest antibiotics, there is 20–40% mortality within 30 days of bacteremia[5] due to antibiotic-resistant strains[6–9].

Passive and active immunization strategies have failed to significantly reduce morbidity and mortality from *S*. *aureus* infections, with few candidates progressing into Phase II and III clinical trials[5, 10]. Therefore, targeting alternate antigens on the *S*. *aureus* cell surface as well as toxins, and the identification of novel immunotherapeutics are still being explored with priority [11–21]. One of the key virulence factors on the surface of *S*. *aureus* is the immunoglobulin (Ig) binding protein SpA[22, 23], a 42-kDa protein composed of five homologous Ig-binding domains, and a C-terminal anchor to the bacterial cell wall. Each Ig binding domain binds the Fcγ region of human IgG 1,2 and 4 with high affinity[24] and also within the Fab region in the case of the human VH3-type IgM and B cell receptor [25]. The only human IgG subclass that does not bind to SpA with a notable affinity is IgG3[26]. Binding of Fcγ on the cell surface of *S*. *aureus* could inhibit opsonophagocytic killing of staphylococci by polymorphonuclear leukocytes [27]. Mutants of *S*. *aureus* lacking SpA are more efficiently phagocytosed *in vitro*, and mutants in infection models have diminished virulence[28].

The present study was designed to isolate and characterize antibodies against *S*. *aureus*, with a focus on SpA as the target antigen, as it is present on both antibiotic susceptible strains as well as multidrug resistant strains such as Methicillin Resistant *Staphylococcus aureus* or “MRSA”. We have identified and isolated several anti-SpA antibodies from healthy human donors who have natural antibodies against SpA, produced these antibodies in mammalian cells in an IgG3 format, and conducted *in vitro* assays to characterize the antibodies. These antibodies bind with high affinity to wild type SpA, opsonize *S*. *aureus* in the presence of other human antibodies, have an Fc capable of binding to Fcγ receptors present on phagocytes, and are able to facilitate the opsonophagocytic killing of both MSSA and MRSA isolates. Finally, we have shown the prophylactic efficacy of one of the antibodies, 514G3, both as a first line treatment, and in combination with vancomycin in a mouse model with MRSA bacteremia.

## Materials and methods

### Ethics statement

Human blood was collected at the South Texas Blood and Tissue Center (STBTC, 6211 IH 10 W., First Park Ten Blvd. San Antonio, TX 78201). Experiments with blood from human volunteers were performed under protocol number PT016. This protocol has been reviewed, approved, and supervised by the Western Institutional Review Board (WIRB). The donors signed a written informed consent prior to donating blood for this study. For the animal studies, 6–8 week old female Balb/C mice were purchased from Charles River Laboratories. Mice were housed and handled in the animal facility at XBiotech USA Inc. in strict accordance with good animal practice; as defined in the federal regulations set forth in the Animal Welfare Act (AWA), the 1996 Guide for the Care and Use of Laboratory Animals of the National Institutes of Health, and our institutional policies for care and handling of mice (Protocol numbers AF005, AF006, AF012, AF013, AF021, and AF023). Mice were kept under standard environmental conditions of temperature and light; and were fed global soy protein-free extruded rodent diet, and water *ad libitum*. The Institutional Animal Care and Use Committee (IACUC) at XBiotech USA Inc. approved the study.

### Blood collection

Blood from healthy donor P656 was collected at STBTC. The blood was stored on cold packs and the plasma and B-cells were isolated within 3 hours of collection.

### B-cell isolation

Unit blood from donors with high titers of anti-SpA antibodies was collected at STBTC. The blood was centrifuged at 1000g for 20min to separate plasma from red blood cells and PBMC. Plasma was collected and processed as described below. PBMC was isolated using ACCUSPIN^™^ tubes (Sigma-Aldrich, A2055) and Ficoll gradient (Ficoll-Paque premium, Sigma-Aldrich; GE17-5442-02) per manufacturer’s recommendation. B-cells were isolated from PBMC by EasySep^™^ Human B Cell Enrichment Kit from Stemcell Technology (19054) according to the manufacturer’s protocol. The quality of the isolated B-cells was determined on a FACS Calibur, and the quantity was determined using a Vi-Cell cell counter.

### RNA isolation

B-cells were pelleted by centrifugation at 300xg for 5 min. 1x10^7^ cells were lysed with TRIZOL^®^ reagent (ThermoFisher Scientific, 15596026) and incubated at room temperature for 5 min. 0.2 ml chloroform (Sigma-Aldrich, 611776) was added per 1 ml of TRIZOL^®^ reagent, and the tubes were shaken vigorously for 15 seconds, and incubated at room temperature for 3 minutes. The samples were centrifuged at 12,000xg for 15 min at 4°C. The RNA was precipitated from the aqueous phase using 1 volume of isopropanol. The RNA was washed with 70% ethanol, dried under vacuum, and re-suspended in nuclease free water.

### cDNA synthesis using SuperScript III^™^

cDNA was synthesized using oligo(dT)_20_, dNTP and SuperScript III using the recommended protocol by the manufacturer (ThermoFisher Scientific, 18080085). The final cDNA was dissolved in nuclease free water and stored at -80°C.

### Polymerase chain reaction to isolate variable heavy and variable light chain sequences

PCRs were done with forward primers that bind to the leader regions of the heavy and light chains; and reverse primers that bind to the hinge region of IgG2 (S3 and S4 Tables) and constant regions of Igk. Based on the data generated by isotyping (not shown) and mass spectrometry (S2 Table), nested PCRs were done on the VH3 subfamily of heavy chain, and VK1 subfamily of light chain amplified products, to add Sfi adapters (5’-GGGCCCAGGCGGCC-3’) on the 5’ end of the light chain, and linker adapters on the 3’ end (5’-GGAAGATCTAGAGGAACCACC-3’). The heavy chain nested PCRs were done to add longlinker on the 5’ end of heavy chain(5’GGTGGTTCCTCTAGATCTTCCTCCCTGGTGGCGGTGGCTCGGGCGGTGGTGGG-3’), and Sfi adapter on the 3’ end of heavy chain (5’-CCTGGCCGGCCTGGCCACTAGT-3’).

Overlap extensions, to generate V_L_-linker-V_H_, were done using 1:1 mixture of the V_L_ and V_H_ PCR fragments in two phases. In the first phase, the mixture was incubated at 94°C for 2 min, followed by 10 cycles of 94°C for 30 s, 54°C for 30 s, and 68°C for 2 min. At the end of Phase 1, an equal mixture of the following forward primers (RSC-Vreg-K1 GAG GAG GAG GAG GAG GAG GCG GGGCCCAGGCGGCCGA, RSC-Vreg-K2 GAG GAG GAG GAG GAG GAG GCG GGGCCCAGGCGGCCGT, and RSC-Vreg-K3 GAG GAG GAG GAG GAG GAG GCG GGGCCCAGGCGGCCGC, and reverse primer, RSC-Rev GAG GAG GAG GAG GAG GAG CCT GGC CGG CCT GGC CAC TAG TG was added to the mixture, and the Phase 2 part of the PCR was run 94°C for 5 min, followed by 25 cycles of 94°C for 30 s, 64°C for 30 s, and 68°C for 2 min. A final extension of 68°C for 10 min was performed.

The V_L_-linker-V_H_ DNA fragments were digested with Sfi, and 5μg of the Sfi cut insert was ligated with 10μg of the Sfi cut pXBPAN1.1 phagemid vector. Ligation mixes were incubated at 16°C for 15 hours, heat inactivated at 65°C for 10 min and the DNA was precipitated using salt and ethanol, and the pellet was dissolved in 100 μl nuclease free water. DNA was electroporated into XL1Blue electrocompetent cells (Agilent Technologies, 200228) to generate the phage library. The library diversity was calculated to be 2.2 x 10^8^ molecules.

### Affinity selection of phage library with WT SpA

Streptavidin conjugated Dynabeads (M-280 Streptavidin, ThermoFisher Scientific, 11205D) were coated with biotinylated SpA (Sigma-Aldrich, P6031). Seven successive rounds of biopanning were performed on the phage library without phage amplifications in between using the following method: the phage library was incubated with SpA coated magnetic beads for 1 hour with rotation. The tube was placed in a magnetic holder and the supernatant was removed from the beads bounds with phage particles. Beads were thoroughly washed 10–15 times with PBS containing 0.5% Tween 20 and the bound phage particles were eluted with Glycine-HCl (pH 2.2). The eluate was neutralized with 1M Tris to reach pH 7.0 and used for the next round of panning. After 7 rounds of panning, phage particles were collected and mixed with XL1Blue cells, plated and the colonies were picked. All clones were analyzed using phage ELISA and the DNA was sequenced.

### Phage purification for ELISA

Phages were prepared from 3 ml overnight cultures as follows. Bacterial clones bearing recombinant phagemids were grown in 1 ml SB medium supplemented with carbenicillin at 50 μg/ml, to a density of 0.5 Optical Units/ml. Cultures were super-infected with helper phage VCSM13 (Agilent Technology, 200251) with ratio: 1 cell: 20 helper phage virions; incubated without shaking for 30 min at room temperature, then incubated for 2 hour at 37°C in a shaker incubator at a speed of 200 rpm. 2ml SB medium supplemented with the following antibiotics were added to the original cultures to bring the final concentration of carbenicillin to 50 μg/ml, tetracycline to 10 μg/ml and kanamycin to 25 μg/ml in 3 ml volume.

Cultures were grown overnight at 37°C. Culture supernatants containing phages of interest were obtained by centrifugation at 2300xg for 5 min at room temperature. Phage particles were precipitated by adding 1 volume of 25mM PEG-8000, 2.5M NaCl to 4 volumes of supernatant, and mixtures were incubated for 1h on ice. Phage pellets were obtained by centrifugation at 16100xg for 15 min, and re-suspended in appropriate volumes of sterile 1X PBS. Phage preparations were clarified from residual cell debris by centrifugation at 16100xg for 5 min. Optical density of the phage preparations was estimated by nanodrop at 269 and 320 nm. Phage physical titers (number of virion/ml) were calculated based on the formula:

$$\text{Physical}\;\text{Titer}\;\left(\text{vir}/\text{ml}\right)=\left[\left({\text{A}}_{269}-{\text{A}}_{320}\right)\;\text{x}\;6\;\text{x}\;10^{16}\right]\;/\;\text{S};$$

where S is the total number of nucleotides in the phage construct.

### Phage ELISA

The physical titer of the phage clones was adjusted to 3–5 x 10^12^ virus/ml in PBS. 30 μl of phage preparation was added to one well of ELISA plate (Corning Inc., 3690) and incubated overnight at 4°C. Next morning, the wells were washed 3X with PBS containing 0.1% Tween 20 (wash buffer). The wells were blocked with 3% BSA in PBS (blocking buffer) for 1 hour at room temperature followed by 6 washes with wash buffer. 10 μg/ml of biotinylated SpA was added to the wells, incubated for 1 hour followed by 6 washes with wash buffer. Finally, Streptavidin HRP was added in 1:1000 dilution in blocking buffer, followed by detection with TNB. Eight clones with high positivity on this ELISA were selected for further characterization.

### Cloning of heavy and light chain variable regions into expression vector

The light and heavy chain sequences were codon optimized for Chinese Hamster Ovarian (CHO) cells, and synthesized as mini genes (GeneArt Gene Synthesis, ThermoFisher Scientific). The heavy chain was sub-cloned into Lonza vectors pEE6.4 (for heavy chain) and pEE12.4 (for light chains) using forward primers containing a Kozak sequence and Lonza specified leader sequence and a homology with the antibody sequences, and the reverse primers with a region of homology with the 3’ end of the heavy and light chain variable regions. The dual gene vector (DGV) was prepared by digesting the pEE6.4 and pEE12.4 with the restriction enzymes PvuI and NotI (New England Biolabs), gel purifying, and re-ligating the two inserts. The DGV was transfected into CHOK1SV cells.

### Transfection of antibodies into CHOK1SV cells

CHOK1SV cells (Lonza Biologics) were cultured in CD CHO medium (Gibco, 10743029) supplemented with 6 mM L-glutamine (Gibco, 25030081). Ten million CHOK1SV cells were transfected with 40 μg of linearized DGV plasmid by electroporation using Bio-Rad Gene Pulser Xcell Total System (Bio-Rad, 1652660). Transfected cells were cultured in CD CHO medium in T175 flasks, and 50μM of MSX (L-Methionine Sulphoximine, Sigma Aldrich, 76078-1G) was supplemented on the day after transfection. The pool of transfected cells was expanded in culture until the cell viability reached above 85%, after which the concentration of MSX was brought down to 25μM. Transfected cells (above 95% viability) were plated in CloneMedia-CHOK1SV semi-solid media (Molecular Devices, K8725) containing CloneDetect, Human IgG (H+L) Specific, Fluorescein, BSA Free (Molecular Devices, K8202) in 6-well plates (Greiner Bio-One, 657185) in a 37°C incubator with 10% CO_2_. Top producing clones were picked by ClonePix2 (Molecular Devices) according to their high FITC 500 ms Sum Total Intensities on Day 14. Selected clones were screened in static and suspension cultures in CD CHO medium for the assessment of productivity. The titer of antibody was analyzed using an Octet RED96 (ForteBio). The 514G3 cell line was the top PA8 antibody-producing clone selected.

### Antibody purification

Mammalian cell culture containing target antibody was centrifuged at 5000xg for 20 minutes. The supernatant was filtered through a 0.22 μm filter and the antibodies were affinity purified on a Protein A column. The antibodies were eluted in 50 mM sodium citrate (pH 3.2), and the pH was neutralized. The antibody was sterile filtered using 0.1 μm filter. The concentration of purified antibody was measured by A280.

### Bacterial strains

NR-46223 (MRSA): *Staphylococcus aureus* strain GA-92; ATCC 25923 (MSSA): *Staphylococcus aureus* subsp. aureus Rosenbach. The bacterial cells were obtained from BEI Resources (ATCC). *S*. *aureus* cells were taken from a frozen glycerol stock and were inoculated onto a single blood agar plate and grown for 20 hours at 37°C. Single colony of *S*. *aureus* was inoculated in a 100ml Tryptic Soy broth and incubated in a shaking incubator (220 rpm) at 37°C for approximately 2 hours until the OD reached ~0.40 at 600 nm. The bacterial suspension was pelleted by centrifugation at 3500xg at 4°C for 10 minutes. The bacteria were washed in 1xPBS and re-suspended in 10 ml of freezing media (10% glycerol in tryptic soy broth). The bacterial stock was frozen in an ethanol/dry ice bath and placed in a −80°C freezer. The concentration (CFU/ml) of the frozen stock was determined by thawing an aliquot of stock, and preparing serial dilutions from 10^−2^ to 10^−10^. The dilutions were plated (100μl) as duplicates on blood agar plates and incubated for 37°C for 16–18 hours. The CFU/ml was determined as follows: CFU/ml = (average # of colonies x dilution factor x 10) and the average for each dilution was used to determine the CFU/ml. For mouse *in vivo* bacteremia studies, on the day of the study, one aliquot of the specific strain was thawed, and appropriate dilutions were made for injection into mice.

### Opsonophagocytosis assay for lead candidate selection

HL60 cells (American Type Culture Collection, CCL-240) were differentiated into phagocytes by supplementing the culture media (IMDM+20% low Ig serum) with 0.8% of dimethylformamide (DMF, ThermoFisher, 20673) for 5 days. *S*. *aureus* were labeled with pHrodo-green (ThermoFisher, P35369) and antibodies with pHrodo-red (ThermoFisher, P36600). *S*. *aureus* (ATCC-25923) were incubated with 50μg/mL of the candidate antibodies for 30min at 37°C. The unbound antibody was removed by centrifugation, and the opsonized bacteria were incubated with differentiated HL60 cells for 30–90 min at 37°C, at a bacterium to cell ratio of 50:1. Phagocytosis was measured by the presence of pHrodo green signal inside the cell (FACSCalibur, BD Biosciences) and the percent of cells with positive signal were plotted on a graph.

### Binding kinetics analysis

Octet Red 96 (ForteBio) was used to select the anti-SpA antibodies with highest affinities after the phage panning using Anti Human Capture (AHC) sensors and 20nM, 40nM, and 80nM SpA. For binding studies to SpA domain variants, Biotin conjugated (Pierce EZ-Link^™^ NHS-PEG4-Biotin, 21330) 514G3 and VH3/IgG3-k isotype control antibodies were prepared at 5μg/mL in MD5xKB (DPBS with 0.05% BSA and 0.02% Tween-20) and dispensed into a black 96-well microplate (200μL/well). SpA (Pierce, 21181), MabSelect Ligand (GE Life Sciences, 28-4018-59), and MabSelect SuRe Ligand (GE Life Sciences, 28-4018-560) were also prepared in MD5xKB and dispensed in 2-fold dilution series (20–0.625 nM of SpA/MabSelect, 200–0.625 nM of MabSelect SuRe for 514G3 and 20–0.625 μM for isotype control). Regeneration buffer (10mM Glycine, pH 1.5), and MD5xKB (neutralization and baseline) were also dispensed. The assay plate was agitated at 1000rpm in the experiment. One column (8 each) of biosensors was pre-hydrated in MD5xKB for 10min. Streptavidin biosensors (ForteBio, 18–5019) were baselined (60 sec) in MD5xKB before and after loading (300 sec) test antibodies (4 for each mAb). The binding kinetics were measured by dipping loaded biosensors in varying concentrations of ligands (1 per ligand and 1 for reference). The interactions were monitored during association and dissociation period, in MD5xKB. Biosensors were regenerated with three cycles of 5 secs in regeneration buffer followed by neutralization. The data were processed with the device accompanied data analysis software (Version: 9.0.0.14). A parallel reference buffer blank was subtracted, and the data were aligned at the y-axis using the baseline and smoothed by Savitzky-Golay filtering. SpA and MabSelect were fit to a 1:1 model while MabSelect SuRe was fit to a 2:1 (heterogenous) model with the larger contributor to binding made by the high affinity interaction.

Affinity measurement using Biacore was performed by Precision Antibody (Columbia, MD). Briefly, antibody was captured on flow cell coated with goat anti-human IgG Fc at a flow rate of 10μl/min. Scouting analysis was first performed using ~10 nM concentration of the analyte (WT SpA; Sigma, P6031) in an assay buffer containing 10 mM HEPES buffer (pH 7.4), 150 mM NaCl, 3mM EDTA, 0.005% P20 (polyoxyethylenesorbitan). The scouting analysis provided a rough K_D_. Full kinetic analysis was performed at a flow rate of 50 μl/min using analyte concentrations of 2, 1, 0.5, 0.25, 0.125, 0.0625 and 0 nM. Chi square (χ2) analysis was carried out between the actual Sensorgram and the sensorgram generated from the BIAnalysis software to determine the accuracy of the analysis. A χ2 value below 1 was highly significant (highly accurate). The equilibrium constant (K_D_) was calculated from the observed ka and kd using the BIAnalysis software.

### 514G3 ELISA with Protein A domain variants

High binding ELISA plate (Corning Inc., 3690) was coated with 2μg/ml WT SpA or MabSelect Ligand or Mab Select Sure Ligand and incubated overnight at 4°C. Next morning, the wells were washed 3X with PBS/0.1% Tween 20 (wash buffer) and blocked with 2% BSA in PBS (blocking buffer) for 1 hour at room temperature. 4 μg/ml of biotinylated 514G3 or VH3/IgG3-k isotype control was added to the wells, incubated for 1 hour followed by 3 washes with wash buffer. Finally, Streptavidin HRP was added in 1:2000 dilution in blocking buffer, followed by detection with TNB at 450nm.

### Fcγ receptor binding assay

*S*. *aureus* cells (ATCC-25923) were incubated with 5μg/ml of 514G3 or VH3/IgG3-k isotype control for 15 min. Following this, 5μg/ml of biotinylated FcϒR1A (CD64A, Sinobiologicals, 10256-H27H-B) was added and incubated for 15 additional minutes. The signal from the bound FcγR1 was detected by Streptavidin-APC (BD bioscience, 349024) on a BD Accuri and FlowJo 10.0.8 was used to plot total number of cells against the median fluorescence intensities in the APC channel. Two different controls were used in this experiment; one without antibody, and the other without antibody and biotinylated FcγR1A, to look for background staining from the biotinylated receptor and/or streptavidin-APC to the *S*. *aureus* cells. In the experiment looking at 514G3 and VH3/IgG3-k isotype control binding to *S*. *aureus* cells, the antibodies were labeled with APC and stained directly to visualize their ability to bind to the surface of the cells.

### Competition assay with human immunoglobulin

1x10^9^ *S*. *aureus* cells (ATCC-25923) were incubated with 5mg of pooled human intravenous immunoglobulin preparation (IVIG, Gammagard, Baxter) for 15 min at 37°C. Following this, 100μg/ml of 514G3 or the VH3/IgG3-k isotype control labeled with APC was added and incubated for 2.5 hrs. Samples were collected at 15min, 1hr and 2.5hrs, and the fluorescent staining was detected on a BD Accuri. The raw data was analyzed using FlowJo 10.0.8 and the APC signal was plotted against the total cell count.

### Confocal microscopy

Raw 264.7 macrophages were cultured on Poly-L-Lysine coated cover slips at a concentration of 5x10^5^ cells/well. Washed CFSE labeled *S*. *aureus* (ATCC-25923, 5x10^6^) cells were incubated with 50μg/ml of 514G3 or VH3/IgG3-k Isotype control labeled with APC for 30 min at 37°C on a shaker prior to their addition to macrophage contained cover-slips. After 30min, the coverslips were washed thrice with PBS to remove extracellular bacteria and fixed with 4% paraformaldehyde. Cells were permeabilized in 0.2% Tween 20 in PBS for 10 min and mounted using prolong Diamond Antifade Mountant and DAPI (ThermoFisher, MAN0010261). Phagocytosis was visualized using a Leica SP5X confocal microscope upon focusing on the center of the macrophages.

### Opsonophagocytic killing assay

For opsonophagocytic killing by human blood, 50 μl of O.D. 0.4 culture of *S*. *aureus* (NR-46223) were pre-incubated with 20 μg/ml 514G3 or VH3/IgG3-kisotype control antibody for 20 min. This mixture was then added to 950 μl of freshly drawn human blood and incubated at 37°C with slow rotation. In parallel, the bacterial culture was plated to obtain the viable bacterial plate counts. For the blood cell plating assay, gentamicin (200 μg/ml) was added after 30 min incubation to kill extracellular bacteria. At 30, 90, and 150 minutes, the 100 μl samples were withdrawn, blood cells were pelleted at 300xg, washed twice with HBSS, and lysed with 1% saponin in PBS. CFUs were determined by plating the dilutions on TSB agar. For the whole blood plating assay, no gentamicin was added. After co-incubating of opsonized *S*. *aureus* and whole blood for 30, 90 and 150 minutes, the samples were withdrawn and lysed with 1% saponin-PBS and CFUs were determined by plating the dilutions on TSB agar.

### Bacteremia animal model

Female Balb/C mice (6–8 weeks of age) were purchased from Charles River Laboratory, NIH, Maryland and were kept at 5 mice per cage with a 12-hr light dark cycle. The mice were fed with global soy protein-free extruded rodent diet and clean water ad libitum. Assessments were carried out twice daily to ensure well-being of all the animals. For the 514G3 efficacy studies, mice were randomized to either 514G3, VH3/IgG3-kisotype control, or vehicle control at 10 per group. *S*. *aureus* sepsis was induced by intravenous injection (i.v.) of 3 × 10^7^ CFUs of MRSA strain NR-46223. Three hours prior to infection, mice in the treatment groups were intravenously administered 10mg of 514G3, and the mice in the control group were administered either 10mg of VH3/IgG3-k isotype control or an equivalent volume of vehicle alone. For vancomycin studies, 0.5 mg of vancomycin was administered via the intraperitoneal route along with 0 or 2.5 mg of 514G3 two hours prior to infection with *S*. *aureus* (NR-46223 i.v.). Clinical signs of illness (physiological and/or behavioral distress) in each mouse were evaluated twice daily for 14 days. This included ruffled fur, reduced mobility, hunched back, panting, signs of hypoxia, anorexia, and death. Animals showing the four signs of distress including hunched back, ruffled fur, excessive panting and more than 25% weight loss were humanely euthanized. Euthanized mice were considered as deaths, as pilot experiments showed that mice with severe illness die before the next time point. The total number of mice in this study were seventy. Of these, twenty-five mice were euthanized and forty-five animals were found dead. The survival rate was monitored over 14 days, and statistical analysis of the survival data was conducted using Kaplan-Meier Survival Analysis with a Mantel-Cox (logrank) test.

## Results

### Isolation of anti-SpA antibody sequences from donor P656

The plasma from donor P656, a healthy donor with natural antibodies against SpA, was separated by centrifugation, and the B-cells were isolated as described in the methods. The plasma was fractionated and the immunoglobulins were subjected to immunoprecipitation on magnetic beads coated with SpA peptides (S1 File, and S1 Table). Since full length WT SpA binds immunoglobulins via their Fc and VH3 framework regions, and prevent the identification of anti-SpA antibodies that bind via the CDRs, the SpA peptides used for immunoprecipitation were designed from regions on the SpA outside the Fcγ and Fab (framework-mediated) binding sites[28]. Mass spectrometric analysis of the human IgGs recovered after immunoprecipitation with SpA peptide coated beads identified high confidence peptides that belonged to VH3 heavy chain and VK1 light chain subfamilies (S1 File and S2 Table). The constant regions were identified to be IgG2 heavy and kappa light chains.

**Fig 1:**
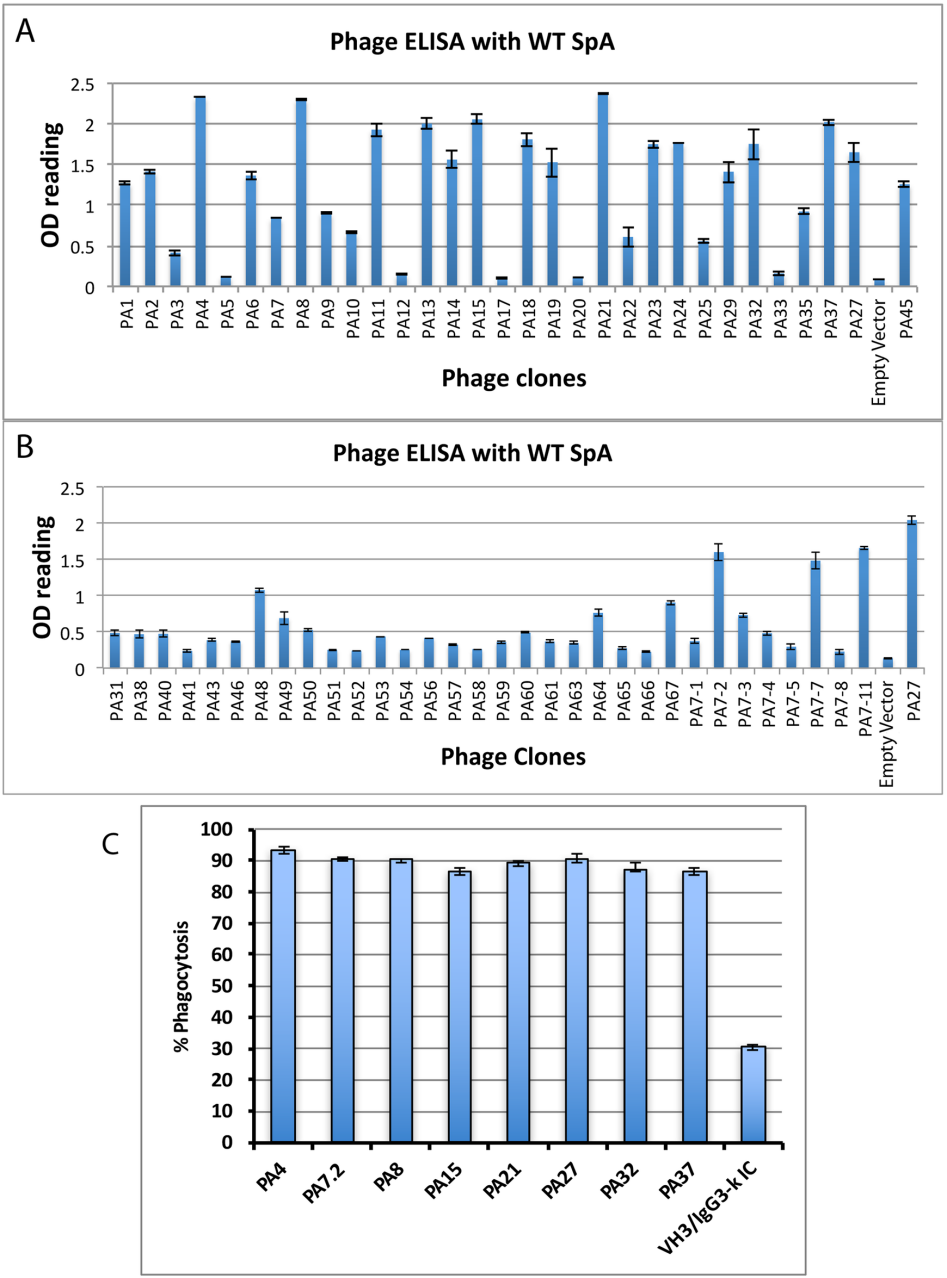
Screening of anti-SpA antibodies using ELISA and Opsonophagocytosis assays. A and B: Phage ELISA to monitor reactivity of the clones after seven rounds of panning: Sixty-two high affinity clones were analyzed by Phage ELISA against SpA from *S*. *aureus*. The x-axis has the different phage clones and the y-axis has the absorbance at 450 nm. The empty vector has a 180 bp stuffer sequence between the two Sfi sites within the phagemid and phage generated with the empty vector is used as a negative control. The error bars show the standard deviation from three replicates. Top eight clones were further tested by opsonophagocytosis assay. C: Anti-SpA antibodies mediate opsonophagocytosis: Differentiated HL60 cells were incubated with opsonized fluorescently stained *S*. *aureus* cells. Increase in fluorescence intensities within cells correlates directly with the internalization of the *S*. *aureus* bacteria through opsonophagocytosis. Greater than 85% of the HL60 cells had phagocytosed CFSE labeled bacteria in the presence of all eight of the anti-SpA antibodies. The background opsonophagocytosis by the HL60 cells in the presence of VH3/IgG3-k isotype control was about 30%. The error bars show the standard deviation from three technical replicates.

In parallel, mRNA was isolated from the B-cells and subjected to cDNA synthesis. Based on the Mass spectrometric results, specific primers were used to amplify VH3 heavy and VK1 light chains from the cDNA (S3 and S4 Tables) and generate an enriched phage library. During phage panning on full-length SpA coated beads, extensive washing was performed with wash buffer at every round to remove low affinity ScFv containing phage particles, and no phage amplification was performed in between the panning rounds to prevent phage growth bias. At the end of seven rounds, ELISA and sequencing analyses were performed after which 62 affinity selected phage clones were analyzed by DNA sequencing. The ELISA results are shown in Fig 1A and 1B. After elimination of phage clones with duplicate sequences, the top eight ScFv sequences: PA4, PA8, PA15, PA21, PA27, PA32, PA37, and PA7-2, were selected for further characterization.

### The antibodies bind SpA via Fab region and promote opsonophagocytosis

Bio-Layer Interferometry (BLI) analysis of the top eight ScFv fragments (PA4, PA8, PA15, PA21, PA27, PA32, PA37, and PA7-2) converted into full-length antibodies of the IgG3 subclass (G3m(b) allotype) showed that the antibodies bound to the WT SpA with picomolar affinity (K_D_ <10^−12^; see S1 Fig). In parallel, since the heavy chain variable regions of all of the antibodies belong to VH3 sub-family, and VH3 framework sequences have been reported to bind SpA outside the classical antigen-binding site of the hypervariable loops[29], an IgG3-kappa isotype control was generated with a VH3 variable region (VH3/IgG3-k) and used in our experiments as the control antibody. The heavy chain of the isotype control has 91.6–93.2% sequence identity with the test antibodies with the differences bring mainly in CDRs. We were unable to detect any binding of VH3/IgG3-k isotype control on Octet up to analyte concentrations of 20μM (not shown), indicating that if any, the binding would have a K_D_ above 10^-6^M.

An opsonophagocytosis assay using differentiated HL-60 cells was performed to test the eight anti-SpA antibodies along with the VH3/IgG3-k isotype control. The results showed comparable and significant phagocytosis, >85%, for all the eight antibodies (Fig 1C), with the background phagocytosis in the VH3/IgG3-k isotype control being about 30%. This background could be due to a combination of spontaneous phagocytosis by the differentiated HL60 cells and non-specific phagocytosis by the VH3/IgG3-k isotype control. Since the top 8 antibodies showed comparable levels of phagocytosis, we analyzed the CHOK1SV pools transfected with the eight antibodies for highest antibody expression. Individual clones picked from PA8, the highest antibody expression pool, were tested for optimal growth in media and antibody production capabilities. PA8 antibody purified from the highest producing cell line was called 514G3, and was characterized further as described below.

### 514G3 binds SpA with high affinity

**Fig 2:**
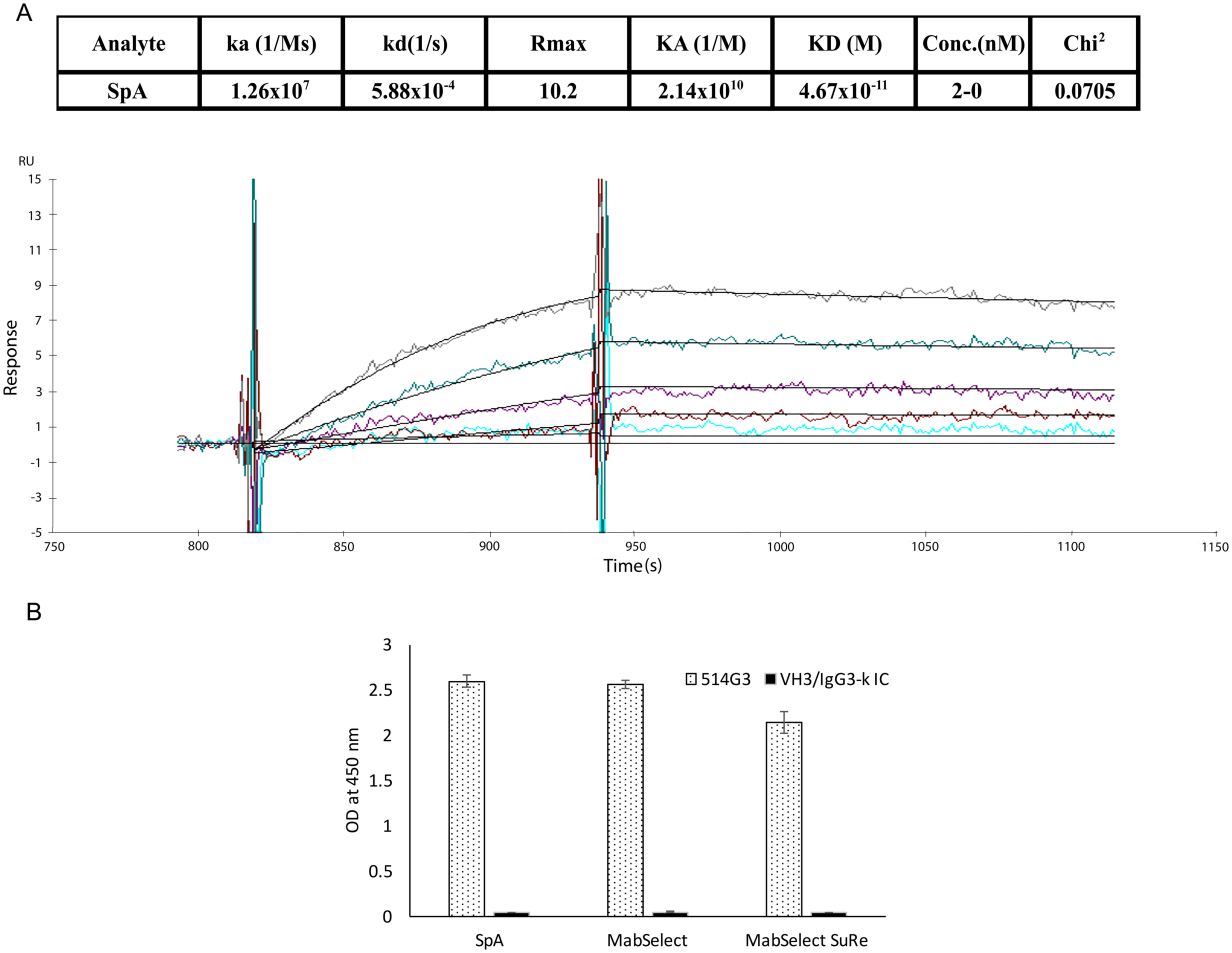
514G3 antibody binds to WT SpA with specificity and picomolar affinity. A. Antigen binding profiles of 514G3 captured on an active flow cell on a Biacore 3000. The colored lines on the sensogram represent the recorded binding response signals at different antigen concentrations from 2-0nM, and the overlaid black lines represent the fitted curves. Binding of SpA to 514G3 was monitored in real time to obtain on (ka) and off (kd) rates. The equilibrium constant (K_D_) was calculated from the observed Ka and Kd and found to be 46.7pM. B. A graph showing the relative binding of 514G3 to SpA, MabSelect and MabSelect SuRe ligands. The three different SpA domain variants were coated on the wells of an ELISA plate, and then probed with biotinylated 514G3 or VH3/IgG3-k isotype control at 4μg/ml, and detected with streptavidin-HRP. The graph shows OD at 450nm on the y-axis and the different SpA domain variants on the X-axis. The error bars show the standard deviation from three replicates.

Because of the high on-target binding affinities of the test antibodies to SpA, BLI analysis used in the initial screen of antibodies only gave a rough estimate of their K_D_ (<10^−12^ M). For high affinity interactions, even the most optimized conditions on an Octet may not yield an accurate K_D_ due to limitations in instrumentation sensitivity for slow dissociation kinetics. Instruments with continuous flow fluidics such as a Biacore are often thought to generate more precise K_D_ in such situations [30]. Therefore, in order to obtain a more precise K_D_ of 514G3 towards WT SpA, we performed Surface Plasmon Resonance (SPR) based analysis on a Biacore 3000. Full kinetic analysis was performed using analyte concentrations from 0 to 2 nM and results are shown in Fig 2A. SpA bound to the antibody 514G3 with an on rate (ka) of 1.26x10^7^ and had an off rate (kd) of 5.88x10^-4^. The calculated K_D_ (M) of the 514G3 antibody towards WT SpA was found to be 46.7x10^-12^M.

### The high affinity binding of 514G3 to SpA is mediated by its CDRs

The classical interaction of SpA is through its five homologous domains, with K_D_ for the Fc regions of human IgG reported to be in the range of 2.6 to 14x10^-6^ M[31] [32]. However, SpA is also reported to bind to VH3 containing Fab fragments via an alternate interaction [33] [34]. Jansson et al [32] have compared the interactions of Fab towards the individual domains of SpA as well as towards a widely used monovalent SpA analog called domain Z. Domain Z is based on domain B of SpA [35], and contains a single glycine to alanine substitution, which abolishes the VH3 containing Fab binding. We hypothesized that if the binding of 514G3 is truly via its CDRs rather than non-specific framework mediated interactions, we would still see high affinity binding of 514G3 towards Z-domain containing ligand. In order to test this, we compared the binding affinity of 514G3 to SpA, MabSelect ligand and MabSelect SuRe ligand using BLI. MabSelect is a recombinant variant of SpA expressed in *E*.*coli* and contains all five binding domains but is devoid of the tail domain known as Xr repeat. On the other hand, MabSelect SuRe ligand is a tetramer of the modified Z domain. S2 Fig shows sensograms and binding affinities of 514G3 towards WT SpA, MabSelect and MabSelect SuRe. As expected, 514G3 has picomolar binding affinity towards both WT SpA and MabSelect, but also maintains picomolar binding affinity towards the Z-domain containing MabSelect SuRe indicating that the binding is specific through the CDRs, rather than the non-specific low affinity binding by the VH3 frameworks. The binding affinities of the isotype control, on the other hand could not be measured by BLI, since there was little binding up to analyte concentrations of 20 μM tested (not shown). Analyte concentrations above 20 μM could not be tested because of technical and instrumentation limitations. The binding of 514G3 to WT SpA, MabSelect and MabSelect SuRe was also tested in ELISA and is shown in Fig 2B. The figure shows that 514G3 binds to all three antigens well, and the isotype control does not bind to any of them, at the antibody concentrations tested (see Methods).

### 514G3 binds to the surface of *S*. *aureus* via Fab and has downstream effector function

**Fig 3:**
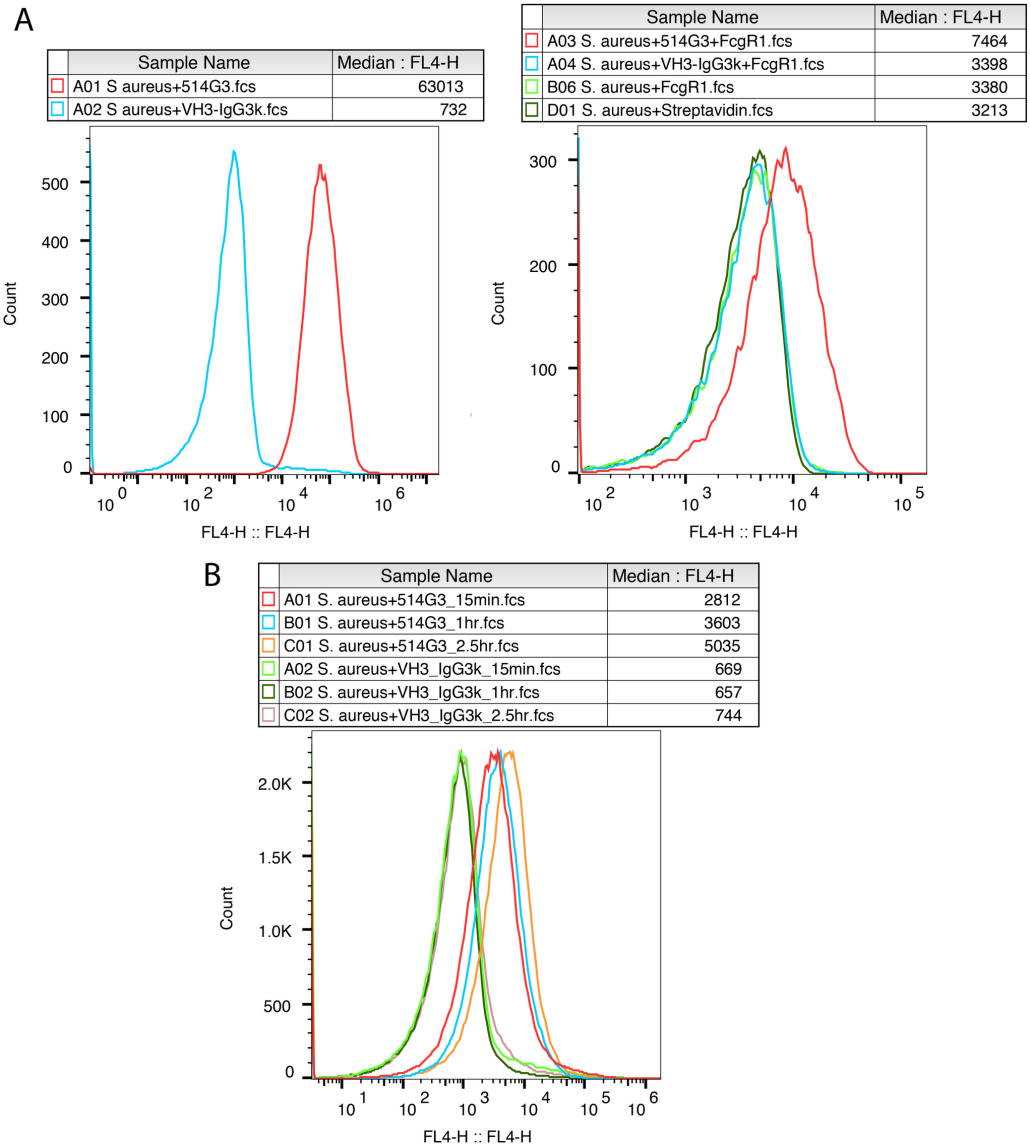
*In vitro* characterization of 514G3. A. 514G3 binds to *S*. *aureus* and leaves the Fc region exposed for FcγR1A receptor binding: 514G3 or VH3/IgG3-k isotype control was incubated with *S*. *aureus* cells for 15 min followed by the addition of biotin labeled FcγR1A. The figures show the overlay of median fluorescence intensities generated by the labeled antibodies or the Fcϒ receptor. There was a specific binding of 514G3 to the *S*. *aureus* cells within 15 min (left panel); and FcγR1A receptor could subsequently bind to the Fc region of 514G3 antibody bound to the surface of *S*. *aureus* (right panel). The right panel also shows two controls where *S*. *aureus* cells were incubated with biotinylated FcγR1A along with streptavidin-APC (light green trace) or streptavidin-APC only (dark green trace) to show that the high background is due to streptavidin-APC. The cells were analyzed on a BD Accuri and the data was analyzed using FlowJo 10.0.8. B. 514G3 is capable of binding to *S*. *aureus* in the presence of pooled human IgG. *S*. *aureus* was pre-incubated with 5mg of pooled human immunoglobulins at 37°C, before the addition of 100 μg/ml of 514G3 or the VH3/IgG3-k isotype control labeled with APC. There was an increase in the binding of 514G3 to *S*. *aureus* cell surface over the 2.5 hours tested. However, the signal with VH3/IgG3-k isotype remained the same over the time points tested. The graph plots the signal in the FL4 (APC) channel versus the total cell count. The median fluorescence intensity values are indicated in the column Median:FL4. The cells were analyzed on a BD Accuri and the data was analyzed using FlowJo 10.0.8, and the data is representative from multiple experimental repeats.

Using flow cytometry, we tested the ability of 514G3 to bind to the surface of *S*. *aureus* (ATCC-25923) via its Fab. We used 514G3 or VH3/IgG3-k isotype control labeled with APC and measured fluorescent intensity in the APC channel. Fig 3A (left panel) shows that 514G3 binds to *S*. *aureus*, whereas the isotype control does not. In order to detect if the bound 514G3 has an exposed Fc that can bind to FcγR1A, we incubated the *S*. *aureus* cells with unlabeled 514G3 or VH3/IgG3-k isotype control followed by addition of biotin tagged FcγR1A receptor, and Streptavidin-APC. Fig 3A (right panel) shows that 514G3 bound to SpA on the surface of *S*. *aureus* can successfully bind to FcγR1A.

The efficacy of an antibody against *S*. *aureus* depends on its ability to bind to SpA in spite of other antibodies bound to it via their Fc. Therefore, next we tested the ability of 514G3 to bind to the surface of *S*. *aureus* in the presence of a pool of human IgGs by flow cytometry. Fig 3B shows that 514G3 successfully binds to *S*. *aureus* pre-incubated with human IgG pool within 15 min, with a continual increase in signal over the subsequent 2.5 hours. At 2.5 hrs of incubation, the signal with 514G3 was about 7-fold higher than the VH3/IgG3-k isotype control, indicating that 514G3 is able to bind to SpA on *S*. *aureus* already bound with human IgGs (Fig 3B).

### Co-localization of 514G3 and *S*. *aureus* in Raw 264.7 cells

**Fig 4:**
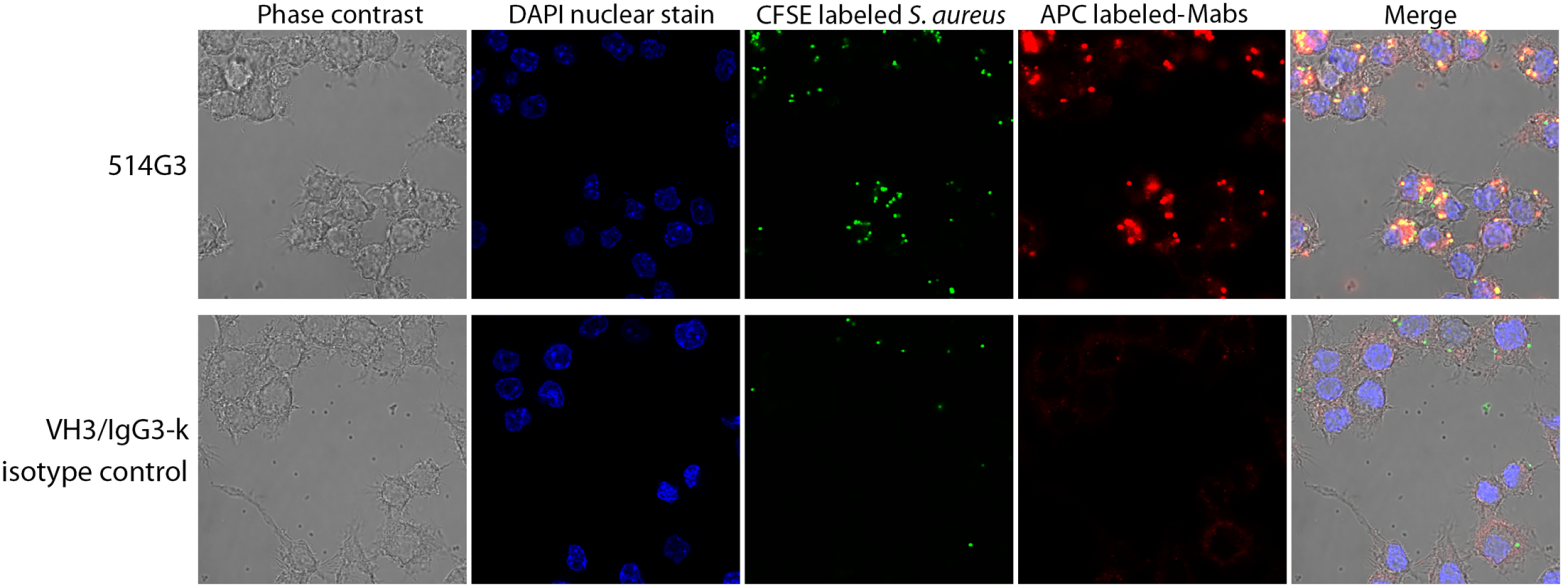
514G3 mediated antibody dependent *S*. *aureus* phagocytosis. A. Visualization of opsonophagocytic uptake of *S aureus* by RAW264.7 cells. *S*. *aureus* were labeled with CFSE (Green), 514G3 (top panel) or VH3/IgG3-k isotype control (bottom panel) was conjugated with APC (Red) and nuclei was stained with DAPI (Blue). The stained cells were imaged on a confocal microscope by focusing on the center of the macrophage to visualize only the bacteria that have been internalized. The top panel shows co-localization of the bacteria and 514G3 as yellow-orange dots inside the cytoplasm of the RAW 264.7 cells. The bottom panel shows that the isotype control does not promote significant opsonophagocytosis as seen by the absence of yellow-orange dots inside the cells.

Next, we monitored the opsonophagocytic capabilities of 514G3 on a confocal microscope. We used RAW 264.7 macrophages rather than HL60 for this study, since they are adherent and do not need DMF based differentiation for phagocytic activity. Fluorescently labeled *S*. *aureus* (green) was incubated with labeled 514G3 (red), and added to coverslips coated with RAW 264.7 macrophages. The nuclei of RAW 264.7 cells were stained with DAPI, in order to distinguish cytoplasmic versus nuclear localization. Sections were visualized on a confocal microscope to focus on to the center of each cell. Fig 4 shows the superimposition of the labeled antibody (red) with the labeled bacteria (green) to generate an orange merge showing co-localization of the two in the cytoplasm of the macrophages (top panel). The VH3/IgG3-k isotype control did not result in significant opsonophagocytosis (bottom panel). This data supports the opsonophagocytic capability of 514G3 after binding to *S*. *aureus*.

### 514G3 antibody supports the opsonophagocytic killing of *S*. *aureus* by human blood cells

**Fig 5:**
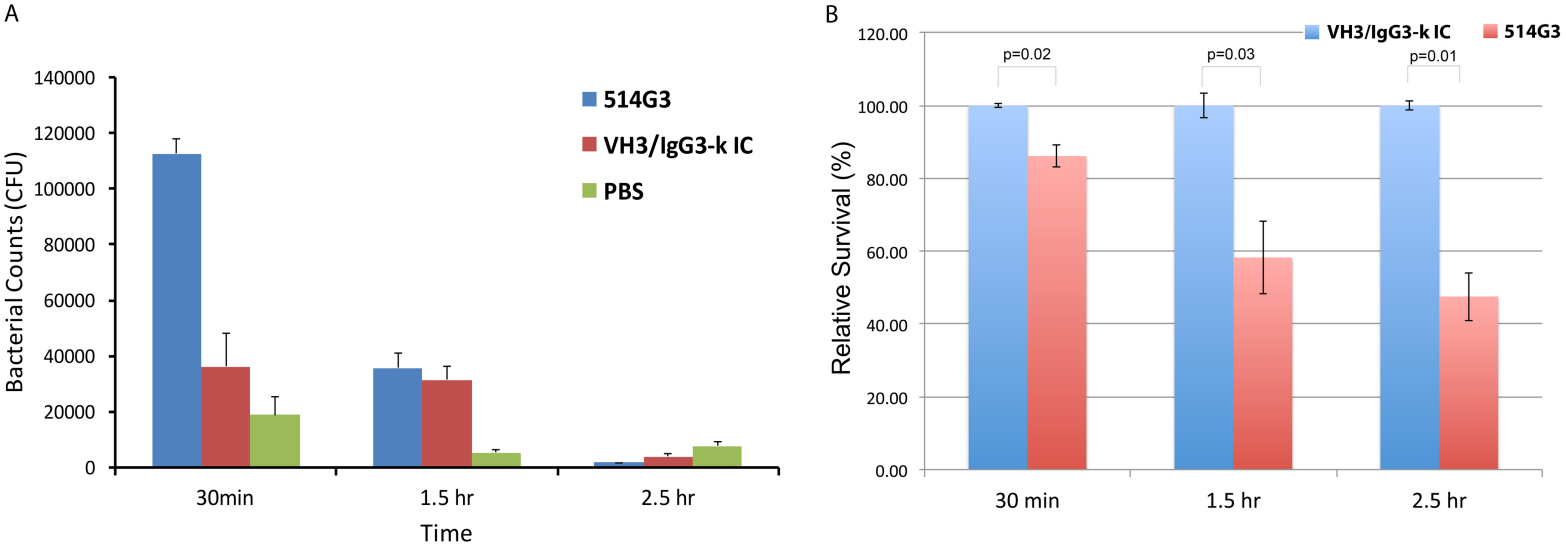
514G3 mediates opsonophagocytic killing of *S*. *aureus*. A. Opsonophagocytic killing of MRSA by human blood cells. Opsonophagocytic killing assay was done using healthy human whole blood. *S*. *aureus* was pre-incubated with either 514G3 or VH3/IgG3-k isotype control, followed by addition to anticoagulated human blood. After 20 min of incubation, the white blood cells were spun down, treated with gentamicin to kill *S*. *aureus* bound on the surface of phagocytes, lysed with saponin and plated at different time points to measure the number of surviving bacteria within the phagocytes. The y-axis shows CFUs in 100μl of lysed sample. The graph shows that >99% of the opsonized bacteria were killed within 2.5 hrs of phagocytosis and the numbers in 514G3 sample, declined over time at a higher rate when compared to either VH3/IgG3-k isotype control or PBS. The error bars show the standard deviation from three replicates. B. 514G3 mediated opsonophagocytosis of MRSA resulted in a reduction of total bacterial load in whole human blood. Anticoagulated human blood was incubated with *S*. *aureus* (NR-46223) in the presence of 514G3 or VH3/IgG3-k isotype control (10μg/ml) for 30 min, 1.5 hr, and 2.5 hr; and survival in the whole blood was measured. Relative survival was calculated at 30 min, 1.5 and 2.5 hrs as the percentage difference in CFU in the 514G3 samples compared with the VH3/IgG3-k isotype control samples. The error bars show the standard deviation from three replicates. The statistical significance was measured using a two-tailed unpaired t-test.

*S*. *aureus* is equipped with various virulence factors that prevent opsonophagocytic killing [36]. In order to evaluate its ability to promote opsonophagocytosis and killing of *S*. *aureus* by human blood cells, we tested 514G3 in an opsonophagocytic killing (OPK) assay alongside the isotype control, as described in the methods. Briefly, *S*. *aureus* cells were incubated with 514G3 or VH3/IgG3-k isotype control before the addition of whole blood. In parallel, the bacterial culture was plated at various dilutions to obtain an actual bacterial plate count. From the bacterial plate counts, the total number of bacteria used in the OPK assay in Fig 5A was calculated to be 13 x 10^6^ CFU, and the total number of bacteria used in the survival assay in the experiment shown in Fig 5B was calculated to be 2.5 x 10^6^ CFU. The results from the OPK assay in Fig 5A shows that incubation of *S*. *aureus* with 514G3 resulted in opsonophagocytosis of the MRSA strain about three-fold over the VH3/IgG3-k isotype control. Upon lysis and plating of the blood cells, sixty-five percent of the opsonized bacteria were killed within 90 min of phagocytosis. The remaining numbers in 514G3 sample declined over time at a higher rate when compared to either VH3/IgG3-k isotype control or PBS. In addition to monitoring bacterial counts over time inside the phagocytes, the relative percentage of total bacteria surviving in whole blood in the 514G3 sample when compared to VH3/IgG3-k isotype control was calculated and plotted in Fig 5B. Relative survival of *S*. *aureus* in the 514G3 treated samples compared to the VH3/IgG3-k isotype control (100%) was 86.12% (p = 0.02) at 30 min, 58.27% (p = 0.03) at 1.5 hrs, and 47.57% (p = 0.01) at 2.5 hours. The bacterial load remaining after 2.5 hrs in the presence of 514G3 antibody was 5.28% of the initial load. These results suggest that 514G3 not only increases phagocytic uptake of *S*. *aureus* by human PMNs, but also increases the rate at which the phagocytosed bacteria are killed with these cells.

### 514G3 protects against lethal challenges with MRSA in a bacteremia model

**Fig 6:**
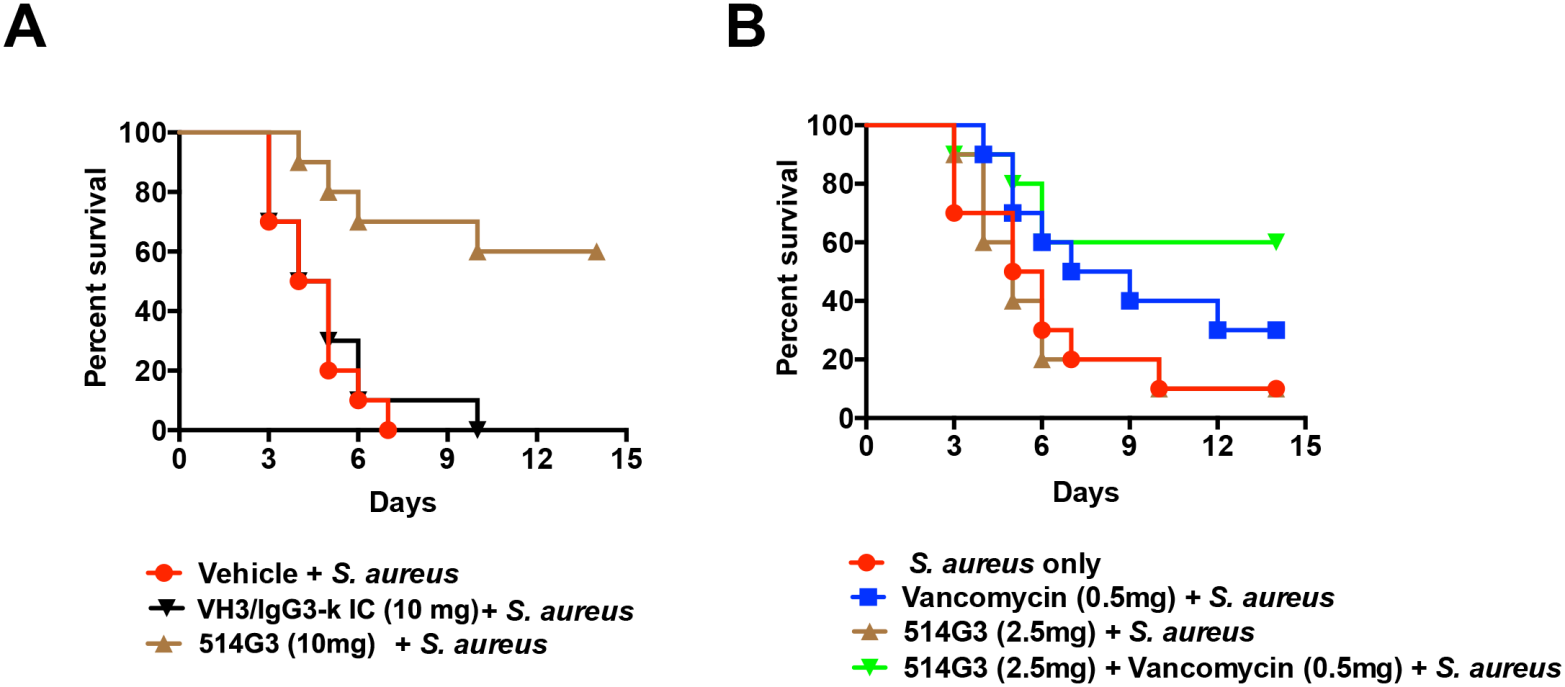
514G3 protects mice from *S*. *aureus* mediated bacteremia. A. Prophylactic administration of 514G3 protects mice against lethal challenge with MRSA in a bacteremia model: BALB/c mice (N = 10) were injected intravenously with 10 mg of 514G3 or VH3/IgG3-k isotype control 3 hours prior to intravenous injection of MRSA (3 × 10^7^ CFUs). The challenged mice were monitored for 14 days, at which point all the remaining mice were sacrificed. The vehicle control mice were injected with vehicle only. At day 14, sixty percent (6/10) from 514G3 group survived, and 0% of mice that received either VH3/IgG3-k isotype control or vehicle only survived the bacterial challenge. The p-values are 514G3 vs. vehicle control p = 0.0006, and 514G3 vs. VH3/IgG3-k isotype control p = 0.0013. B. Effect of 514G3 in combination with vancomycin: Female Balb/c mice (10 per group) from Charles River were injected with one of the following: (i) 0.5 mg of vancomycin via intraperitoneal route, (ii) sub-efficacious dose of 514G3 (2.5 mg) via intravenous route or (iii) 0.5 mg of vancomycin and 2.5mg of 514G3 two hours prior to infection with MRSA (NR-46223 at 2 X 10^7^ CFU i.v.). The control mice received only vehicle. The mice were observed for 14 days at which point all the remaining mice were sacrificed. At day 14, 10% of mice in the no treatment group and sub-efficacious dose of 514G3 survived (1/10), 30% of mice in vancomycin only survived, but 60% of mice in the vancomycin plus 514G3 survived indicating an additive effect due to co-treatment of 514G3 and vancomycin (p = 0.021 compared to antibody only group).

Fatality rates due to *S*. *aureus* bacteremia have decreased only modestly in the recent years. Persistent bacteremia after initiation of appropriate therapy is fairly common in patients, particularly if the infection is due to MRSA [37]. Protective efficacy of 514G3 was investigated in the *S*. *aureus* bacteremia model induced by intravenous injection (i.v.) of 3 × 10^7^ CFUs of MRSA (NR-46223). Due to the predominance of SpA on the surface of *S*. *aureus* as well as released into the bloodstream, and the high load of bacterial cells needed to cause sepsis in mice, we decided to use the maximum feasible dosing via i.v. of 514G3 in this experiment, which was 200 μl of a 50 mg/ml solution. Mice were treated with 514G3 at 10 mg per mouse 3 hours prior to MRSA infection. Control mice were treated with 10 mg of VH3/IgG3-k isotype control or vehicle alone. The mice were monitored for 14 days, after which all remaining mice were sacrificed. Statistical analysis of the animal data was conducted using Kaplan-Meier Survival Analysis with a Mantel-Cox (logrank) test. Fig 6A shows that on day 14, sixty percent (6/10; p = 0.0006 compared to vehicle alone, p = 0.0013 compared to VH3/IgG3-k isotype control) with a 10 mg 514G3 dose survived, and none of the mice that received VH3/IgG3-k isotype control or vehicle alone survived the bacterial challenge.

### Effect of 514G3 in combination with vancomycin

Vancomycin is a standard antibiotic used to treat MRSA bacteremia [38]. Therefore, we tested the effect of co-administration of vancomycin and 514G3 in a mouse MRSA model. We first performed a dose dependent titration of vancomycin and 514G3 to find a sub-efficacious dosage in our mouse model (not shown). This dose was found to be 0.5mg of vancomycin and 2.5 mg of 514G3. Female Balb/c mice (10 per group) from Charles River were injected with 0.5 mg of vancomycin via intraperitoneal route, along with 2.5 mg of 514G3 via i.v. route two hours prior to infection with MRSA (NR-46223 at 2 x 10^7^ CFU i.v.). The mice were observed for 14 days. At day 14, only 10% of the 2.5mg 514G3 treated mice survived, 30% of the vancomycin treated mice survived, but 60% of mice treated with a combination of 2.5mg 514G3 plus 0.5mg vancomycin survived (Fig 6B; p = 0.021 compared to antibody only group), suggesting an additive effect between vancomycin and 514G3. This data indicates that sub-efficacious doses of 514G3 can rescue animals from MRSA mediated bacteremia, when co-treated with sub-optimal dose of vancomycin.

## Discussion

Despite the availability of effective antibiotics, the most serious staphylococcal infections are associated with high mortality[39]. The emergence of antibiotic-resistant strains, and the increase in susceptible and immunocompromised elderly populations, represents a rationale for developing an anti-*S*. *aureus* antibody therapy[40, 41]. The host immune response depends primarily on innate immunity, but is also supported by anti-staphylococcal adaptive humoral immunity. This idea is augmented by the increased frequency of staphylococcal infections among individuals that have low antibody titers (hypo and agammaglobulinemia) and neutrophil function[42]. This indicates that antibody and complement mediated opsonization is required for the elimination of *S*. *aureus* by humans[43].

A few studies have investigated the human immune response to *S*. *aureus* in apparently healthy individuals. One such study[44] analyzed antibody responses against multiple *S*. *aureus* antigens in healthy individuals and acute-phase patients with documented staphylococcal infection. Their results show that healthy individuals can have high levels of circulating anti-staphylococcal antibodies. Their data also indicate that anti-staphylococcal IgG levels can differ greatly, amounting to 0.1 to 3% of total serum antibodies. They also showed that the heterogeneity in antibody levels is obvious in healthy adolescents aged 13 to 15 years, suggesting that antibody levels are already established during childhood.

Twenty percent of the human population are persistent carriers of *S*. *aureus*[45]. Persistent carriers have a 3-fold higher risk of acquiring *S*. *aureus* bacteremia compared to non-carriers[46–49]. However, the risk of death due to bacteremia in persistent carriers are significantly lower than non-carriers[47, 49]. The explanation proposed for this observation is that long-term exposure to colonizing strains in persistent carriers may have allowed their body to develop antibodies that protect them from bacteremia related death. It has also been shown that serum Ig preparations can neutralize toxins from *S*. *aureus* in *in vitro* assays[50, 51].

We have isolated eight different anti-SpA antibodies from a single healthy individual, all of which have picomolar binding affinity towards the protein, indicating that the anti-Staphylococcal response in healthy humans is polyclonal. We have also harnessed the natural ability of human IgG3 Fc to not bind SpA, but retain the high affinity to Fcγ receptors, and cloned and produced these antibodies with IgG3 constant regions. These antibodies are able to bind SpA on the surface of *S*. *aureus*, and their Fc region bind Fcγ receptors on the phagocytes successfully. The lead antibody, 514G3, when administered in a single dose of 10mg/mouse, is able to protect mice from a lethal challenge of MRSA. The reason for the high dosage, we believe, is because bacteremia is established in BALB/c only by injecting with very high CFUs of *S*. *aureus* cells. In our experiments, this was 3 × 10^7^ CFUs. Since SpA is not only one of the most abundant cell surface proteins [52], but is also released from the surface of the bacteria during growth[53, 54], a high concentration of antibody is needed in order to neutralize the SpA moieties present both on the surface and in the bloodstream. Anti-infective antibodies are typically used at 10-50mg/kg doses in humans [55–58]. The 10mg dosage in a mouse, when adjusted to the human body surface area to volume ratio is about 40mg/kg body weight, in the range of the already approved anti-infective antibodies [59].

514G3 has a heavy chain variable region that belongs to VH3 sub-family. One could question if this binding is attributed to the non-specific interactions between VH3 framework regions and SpA rather than the CDRs. This is unlikely because, ELISA and binding affinity analyses with SpA domains variants, and the picomolar binding affinity of 514G3 towards MabSelect SuRe, a Z-domain containing ligand, suggests that the specific binding seen between 514G3 and SpA has CDR involvement. Framework sequence analysis also has shown that phage clones PA20 and PA7-8 had an almost identical framework usage as PA8, however they were non-binders in the SpA ELISA (not shown).

In this paper, we have also shown that 514G3 acts additively with vancomycin therapy. 514G3, at sub-efficacious dosing was able to successfully rescue animals, when co-treated with sub-inhibitory doses of Vancomycin. This is very relevant because *S*. *aureus* strains have an ability to modulate the surface expression of SpA in response to exposure of sub-inhibitory concentrations of antibiotics[60, 61]. Given the high mortality and morbidity of severe *S*. *aureus* infections and the emergence of new strains, one could co-administer 514G3 along with vancomycin to overcome *S*. *aureus* load in the patient. This could also potentially have implications in reducing the burden of antibiotic resistance in the future.

In summary, this work has demonstrated that healthy humans have neutralizing anti-SpA antibodies. These antibodies, when isolated and cloned into an IgG3 background, are successfully able to bind to both MRSA and MSSA strains of *S*. *aureus* via their Fab regions with high affinities, leaving the Fc exposed for opsonophagocytosis by phagocytes. We have also demonstrated that these antibodies can successfully rescue mice from MRSA challenge, and can be used either as a mono therapy, or as a co-therapy along with vancomycin. Further study of the 514G3 in the context of other *S*. *aureus* infection models, such as skin, soft tissue, and pneumonia models, and co-administration with other relevant antibiotics will help predict its success in immune-therapeutics.

## Supporting information

S1 File Supporting information methods. (PDF)

S1 Fig Bio layer interferometry analysis of the affinity of the top eight anti-SpA antibodies to WT SpA. (PDF)

S2 Fig Bio layer interferometry analysis of the affinity of 514G3 to SpA domain variants. (PDF)

S1 Table Sequences of the five short SpA peptides used in the initial screening of healthy donor plasma. (PDF)

S2 Table Peptide sequences identified by immunoprecipitation of anti-SpA antibodies from donor P656 followed by mass spectrometric analysis. (PDF)

S3 Table Sequences of forward (F1-F32) and reverse (HC-R) primers used to amplify heavy chain variable regions from the cDNA of B cells from donor P656. (PDF)

S4 Table Sequences of forward (K1-K16) and reverse (HC-R) primers used to amplify kappa chain variable regions from the cDNA of B cells from donor P656. (PDF)
