# Supplementary material for: A natural human monoclonal antibody targeting Staphylococcus Protein A protects against Staphylococcus aureus bacteremia
Source: PLoS One. 2018 Jan 24;13(1):e0190537. doi: 10.1371/journal.pone.0190537 (PMC5783355; doi:10.1371/journal.pone.0190537)
Supplement: S1 File — (PDF) [file pone.0190537.s001.pdf]

Supporting information

Methods:

Plasma fractionation

Following the isolation of plasma from a unit of human blood containing SpA-reactive antibodies, the plasma was filtered through a 0.2 µm membrane, and loaded onto a XK-50/20 chromatography column containing approximately 100 mL of MAbSelect affinity chromatography resin (GE Healthcare). Human IgG (subclasses 1, 2 and 4) were eluted over a 3-column volume linear gradient of 0.1 M glycine-HCl buffer pH 2.5 and then pH neutralized with 0.1 volumes of 1.0 M Tris-HCl pH 8.5.

Streptavidin-conjugated Dynabead M280 magnetic particles (Life Technologies) were incubated with PEG4-biotinylated SpA peptides (Supplementary Table 1) at a ratio of 2.0 µg of peptide per milligram of magnetic beads. Immunoprecipitations were performed by adding 1.6 mg of peptide conjugated M280 magnetic beads to 50 mL of affinity-purified human IgG, followed by a 60-minute incubation. The beads were washed to release any non-specific protein binding (3X Tris-Buffered Saline containing 0.05% v/v Tween 20; 1X Tris-Buffered Saline). Antibodies were eluted using 6.0 M glacial acetic acid, frozen at -80°C and dried by centrifugation vacuum evaporation.

Individual antibodies within the anti-Protein A oligoclonal response were separated by two-dimensional SDS-PAGE. The first dimension was performed under non-reducing conditions on a pH 7-10 11 cm IPG strip (Bio-Rad Laboratories Inc.). The second dimension (reducing SDS-PAGE) was performed to isolate the heavy and light chain subunits from each individual antibody, separated by isoelectric focusing, and visualized by coomassie blue staining. Heavy and light chain subunit pairs were excised from the polyacrylamide gel for LC-MS/MS analysis.

Mass spectrometric analysis

The individual heavy and light chain subunit pairs were characterized through *de novo* LC-MS/MS amino acid sequencing. Briefly, in-gel tryptic digestions were performed on each of the isolated subunits. Following peptide extraction from the gel pieces, the samples were cleaned using disposable micro-scale reverse phase (C18) ZipTip chromatography and analyzed by RPLC-MS/MS on a Waters Micro-QTof mass spectrometer. *De novo* sequencing was performed with MassLynx BioLynx peptide sequencing analysis software.
