# Supplementary material for: A natural human monoclonal antibody targeting Staphylococcus Protein A protects against Staphylococcus aureus bacteremia
Source: PLoS One. 2018 Jan 24;13(1):e0190537. doi: 10.1371/journal.pone.0190537 (PMC5783355; doi:10.1371/journal.pone.0190537)

S1 Fig: Bio Layer Interferometry analysis of the affinity of the top eight anti-SpA antibodies to WT SpA: Antigen binding profiles of the top eight anti-SpA antibodies using Bio-Layer Interferometry. The colored lines on the sensogram represent the recorded binding response signals at an antigen concentration of 40nM, and the overlaid red lines represent the fitted curves. The data was aligned at the y-axis using the baseline and smoothed by Savitzky-Golay filtering. The equilibrium constant (K_D) was calculated from the observed Ka and Kd using the device accompanied data analysis software.

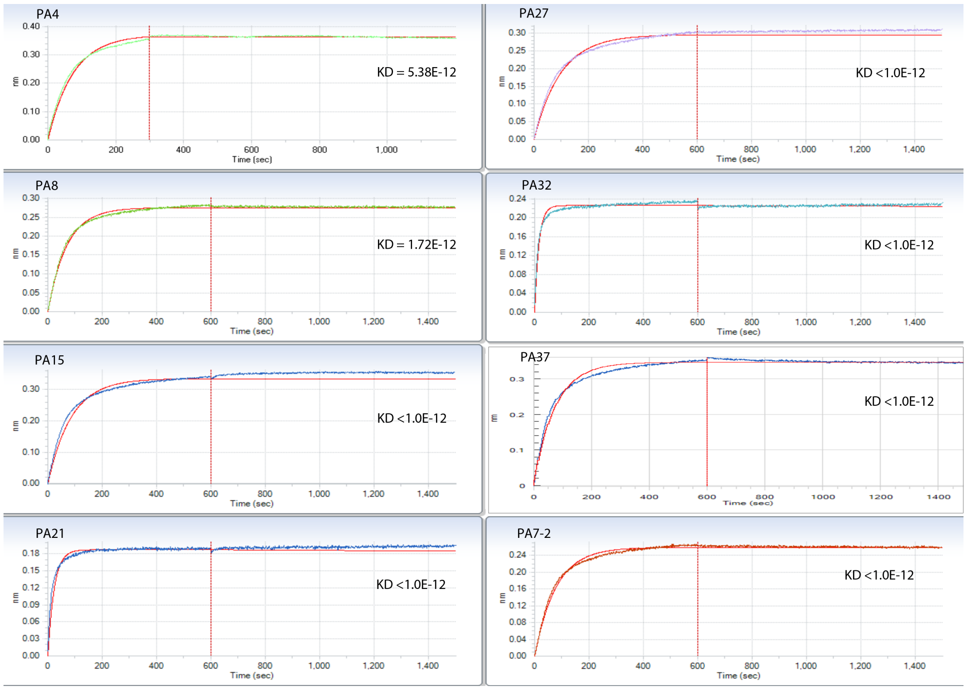

Supplement: S1 Fig — (PDF) [file pone.0190537.s002.pdf]
