# Supplementary material for: A natural human monoclonal antibody targeting Staphylococcus Protein A protects against Staphylococcus aureus bacteremia
Source: PLoS One. 2018 Jan 24;13(1):e0190537. doi: 10.1371/journal.pone.0190537 (PMC5783355; doi:10.1371/journal.pone.0190537)

S2 Fig: Bio Layer Interferometry analysis of the affinity of 514G3 to SpA domain variants: Sensograms showing the relative binding of 514G3 to SpA, MabSelect and MabSelect SuRe ligands. 5μg/ml of biotinylated antibody was loaded onto streptavidin sensors, and binding response signals at antigen concentrations of 20-0.625 nM (for WT SpA and MabSelect SuRe) or 200-0.625nM (for MabSelect SuRe) was recorded. The data was aligned at the y-axis using the baseline and smoothed by Savitzky-Golay filtering. The equilibrium constant (K_D) was calculated from the observed Ka and Kd using the device accompanied data analysis software.

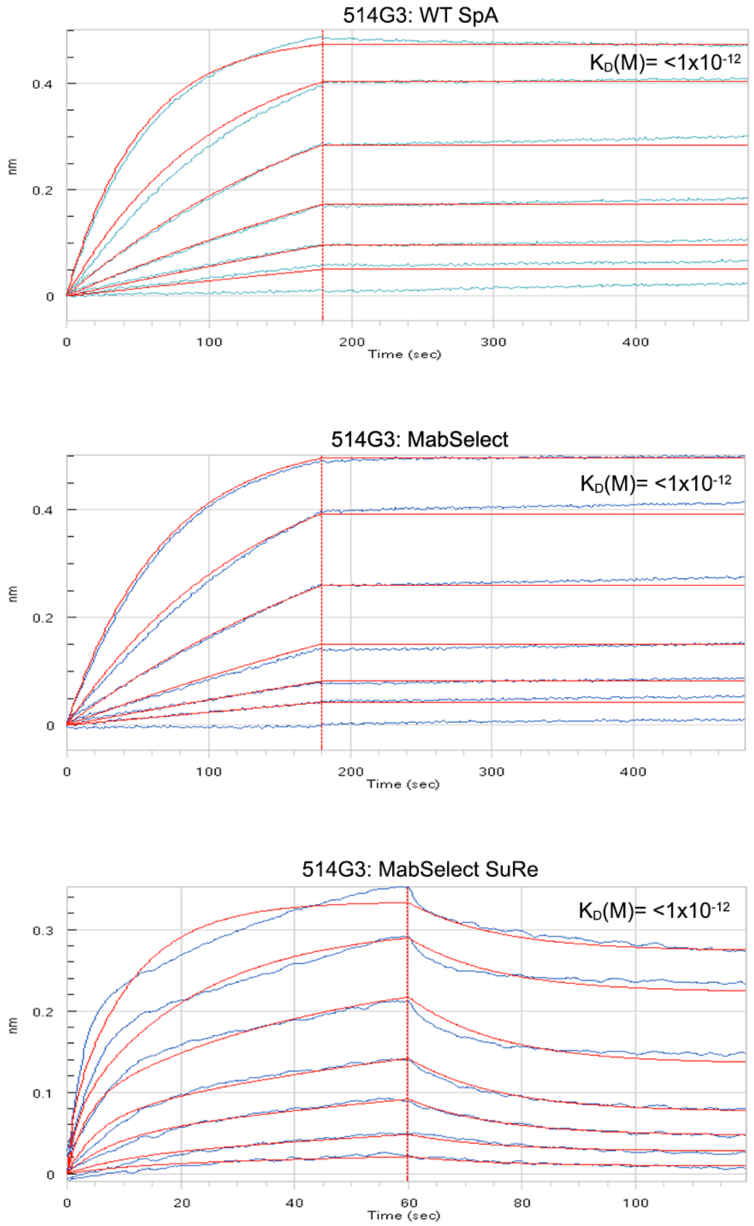

Supplement: S2 Fig — (PDF) [file pone.0190537.s003.pdf]
