# Supplementary material for: A natural human monoclonal antibody targeting Staphylococcus Protein A protects against Staphylococcus aureus bacteremia
Source: PLoS One. 2018 Jan 24;13(1):e0190537. doi: 10.1371/journal.pone.0190537 (PMC5783355; doi:10.1371/journal.pone.0190537)
Supplement: S1 Table — (PDF) [file pone.0190537.s004.pdf]

S1 Table: Sequences of the five short SpA peptides used in the initial screening of healthy donor plasma. Peptides 1 through 4 are located within the five immunoglobulin binding domains, and Peptide 5 is located within the Xr repeat sequence.

Name	Sequence
Peptide # 1	GEAKKLNESQAPKADNNFNKE
Peptide # 2	PNLNEEQRNGFIQSLK
Peptide # 3	AEAKKLNDAQAPKADNKFNKE
Peptide # 4	KEILAEAKKLNDAQAPKEED
Peptide # 5	KPGKEDNKKPGKEDGNKPGK
