# Supplementary material for: A natural human monoclonal antibody targeting Staphylococcus Protein A protects against Staphylococcus aureus bacteremia
Source: PLoS One. 2018 Jan 24;13(1):e0190537. doi: 10.1371/journal.pone.0190537 (PMC5783355; doi:10.1371/journal.pone.0190537)
Supplement: S2 Table — (PDF) [file pone.0190537.s005.pdf]

S2 Table: Peptide sequences identified by immunoprecipitation of anti-SpA antibodies from donor P656 followed by Mass Spectrometric analysis.

Peptide Sequence	Method of Sequencing	IgG Origin
V A G I/L P A P I/L E K	*De novo* unaided Sequencing	G & L unique to IgG2 subclass
S T S E S G G A M G V I/L V K	Mascot Identified Sequence - Confirmed by *de novo* Sequencing	E & S unique to IgG2 subclass
G I/L E W V A N S N Y G S N K	Mascot Identified Sequence - Confirmed by *de novo* Sequencing	GLEWVA & GSNK unique to subfamilies VH3-3-30, 3-30.3, 3-30.5 and 3-3-33
V A T V S P L R	*De novo* unaided Sequencing	V, T & SPL unique to kappa subclass
D I Q M T Q S P S S L S A V G D R	Mascot Identified Sequence - Confirmed by *de novo* Sequencing	DIQMTQSPSSLSASVGDR unique to a subset of VKI subfamily
**NOTE: Underlined text signifies residues which were unique to the identified sub-class/family**		
