# Supplementary material for: A natural human monoclonal antibody targeting Staphylococcus Protein A protects against Staphylococcus aureus bacteremia
Source: PLoS One. 2018 Jan 24;13(1):e0190537. doi: 10.1371/journal.pone.0190537 (PMC5783355; doi:10.1371/journal.pone.0190537)
Supplement: S3 Table — (PDF) [file pone.0190537.s006.pdf]

S3 Table: Sequences of forward (F1-F32) and reverse (HC-R) primers used to amplify heavy chain variable regions from the cDNA of B cells from donor P656. The forward primers are located within the leader sequence, and the reverse primers are located within the CH2 domains of the constant region of the heavy chains.

Primer Name	Sequence
F1 (VH1)	ATG GAC TGG ACC TGG AGG ATC CTC TTC
F2 (VH1)	ATG GAC TGG ACC TGG AGG ATC CTC TTT TTG
F3 (VH1)	ATG GAC TGG ACC TGG AGC ATC CTT TTC
F4 (VH1)	ATG GAC TGC ACC TGG AGG ATC CTC
F5 (VH1)	ATG GAC TGG ACC TGG AGA ATC CTC TTC TTG
F6 (VH1)	ATG GAC TGG ACC TGG AGG GTC TTC
F7 (VH1)	ATG GAC TGG ATT TGG AGG ATC CTC TTC TTG
F8 (VH2)	ATG GAC ACA CTT TGC TCC ACG CTC
F9 (VH2)	ATG GAC ACA CTT TGC TAC ACA CTC CTG
F10 (VH3)	ATG GAA TTG GGG CTG AGC TGG G
F11 (VH3)	ATG GAG TTG GGA CTG AGC TGG ATT TTC CTT
F12 (VH3)	ATG GAG TTT GGG CTG AGC TGG GTT TTC CTT
F13 (VH3)	ATG GAG TTG GGG CTG AGC TGG
F14 (VH3)	ATG GAG TTT GGG CTG AGC TGG ATT
F15 (VH3)	ATG GAG TTT GGG CTG AGC TGG GTT TT
F16 (VH3)	ATG GAA CTG GGG CTC CGC TG
F17 (VH3)	ATG GAG TTT GGG CTG AGC TGG C
F18 (VH3)	ATG GAG TTT GGG CTG AGC TGG GTT
F19 (VH3)	ATG GAG TTT GGA CTG AGC TGG GTT T
F20 (VH3)	ATG GAG TTG GGG CTG TGC TGG
F21 (VH3)	ATG GAG TTT GGG CTT AGC TGG GTT TTC
F22 (VH3)	ATG GAG TTT TGG CTG AGC TGG GTT TTC
F23 (VH3)	ATG ACG GAG TTT GGG CTG AGC TG
F24 (VH4)	ATG GAG TTT GGG CTG AGC TGG G
F25 (VH4)	ATG AAA CAC CTG TGG TTC TTC CTC CTG
F26 (VH4)	ATG AAA CAC CTG TGG TTC TTC CTC CTC CTG
F27 (VH4)	ATG AAG CAC CTG TGG TTC TTC CTC CTG
F28 (VH4)	ATG AAA CAT CTG TGG TTC TTC CTT CTC CTG
F29 (VH4)	ATG AAG CAC CTG TGG TTT TTC CTC CTG
F30 (VH5)	ATG GGG TCA ACC GCC ATC CTC
F31 (VH5)	ATG GGG TCA ACC GCC ATC CTT G
F32 (VH6)	ATG TCT GTC TCC TTC CTC ATC TTC CTG C
HC-R (IgG2)	CGG TGG GCA CTC GAC ACA ACA TTT GCG CTC
