# Supplementary material for: A natural human monoclonal antibody targeting Staphylococcus Protein A protects against Staphylococcus aureus bacteremia
Source: PLoS One. 2018 Jan 24;13(1):e0190537. doi: 10.1371/journal.pone.0190537 (PMC5783355; doi:10.1371/journal.pone.0190537)
Supplement: S4 Table — (PDF) [file pone.0190537.s007.pdf]

S4 Table: Sequences of forward (K1-K16) and reverse (HC-R) primers used to amplify kappa chain variable regions from the cDNA of B cells from donor P656. The forward primers are located within the leader sequence, and the reverse primers are located within the constant region of the kappa light chain.

Primer Name	Sequence
K1 (VK1)	ATGGACATGAGGGTCCCCGC
K2 (VK1)	ATGGACATGAGGGTCCCTGCTCAG
K3 (VK1)	ATGGACATGAGAGTCCTCGCTCAGC
K4 (VK1)	ATGGACATGAGGGTCCTCGCTCAG
K5 (VK1)	ATGGACATGAGGGTGCCCGC
K6 (VK1)	ATAGACATGAGGGTCCCCGCTCAG
K7 (VK2)	ATGAGGCTCCCTGCTCAGCTCC
K8 (VK2)	ATGAGGCTCCTTGCTCAGCTTCTGG
K9 (VK3)	ATGGAAACCCCAGCGCAGCTTC
K10 (VK3)	ATGGAAGCCCCAGCGCAGCT
K11 (VK3)	ATGGAAGCCCCAGCTCAGCTTCT
K12 (VK3)	ATGGAACCATGGAAGCCCCAGC
K13 (VK4)	ATGGTGTTGCAGACCCAGGTCTTCATTTC
K14 (VK5)	ATGGGGTCCCAGGTTCACCTCC
K15 (VK6)	ATGTTGCCATCACAACTCATTGGGTTTCTG
K16 (VK6)	ATGGTGCCCTCGCTGCTCTTTC
R Primer	ACCCGATTGGAGGGCGTTATCCACCT
